# EUGHS NEWS

**DOI:** 10.7189/jogh.06.010204

**Published:** 2016-06

## Council of Science Editors’ award for JoGH’s Editor–in–Chief Ana Marušić

Our Editor–in–Chief Prof. Ana Marušić won the prestigious Meritorious Award from the Council of Science Editors. Prof. Marušić received the Meritorious Award from the Council of Science Editors (CSE) at its 2016 Annual Meeting in Denver, Colorado, USA. The CSE is an international membership organization for editorial professionals publishing in the sciences, serving the scientific, scientific publishing, and information science communities. The Meritorious Award is the CSE’s highest award, recognising those who embrace the purpose of the CSE: improving scientific communication through the pursuit of high standards in all activities connected with editing. CSE also aims to foster networking, education, discussion, and exchange, and acts as a resource on current and emerging issues in the communication of scientific information.

Ana Marušić joins such previous recipients as ORCID, COPE, CrossRef, and her fellow EQUATOR Network steering group member Doug Altman. Prof. Marušić has been active in the publishing and editing world throughout her career. She was the Editor–in–Chief of the Croatian Medical Journal for 20 years and is the Founder and Co–editor–in–Chief of the *Journal of Global Health* (JoGH). She is a past president of both the World Association of Medical Editors and the CSE, and is vice president of the European Association of Science Editors.

Ana Marušić is Professor of Anatomy and Chair of the Department of Research in Biomedicine and Health at the University of Split, School of Medicine in Croatia. She is also a Visiting Professor at the Centre for Global Health Research at the University of Edinburgh. She has been involved with the EQUATOR Network since 2010. Whilst her biomedical research focuses on the interactions between the immune and bone systems, her research interests also include peer review and research integrity, and she is committed to research quality, integrity, and transparency. To this end, she founded the Croatian branch of the Cochrane Collaboration and created the first Croatian public registry of clinical trials.

## Thomson Reuters lists our Editors–in–Chief Harry Campbell and Igor Rudan among “The World’s Most Influential Scientific Minds in 2015”

In their annual publication that tries to identify the scientists amongst the estimated 9 million researchers in the world whose work has earned distinction in the eyes of the scientific community, Thomson Reuters – the large international publishing company behind the most respected scientific citation index, *Web of Science* – identifies a tiny fraction of the authors whose work has consistently resulted in an outsized influence in the form of citations from fellow scientists. Each year the company produces a list of the leading such hundred authors in 21 different areas of science, who are officially designated as “Highly Cited Researchers”. Inclusion to this global rank of researchers is based on the number of papers that have been among the 1% most cited in their respective fields in the previous 10 years. This year, this highly prestigious list has included two of JoGH’s Editors–in–Chief: Prof. Harry Campbell and Prof. Igor Rudan, who have both been selected based on their research in the field of Molecular Biology and Genetics in the previous decade.

## The Royal Society of Edinburgh elected the JoGH’s Editor–in–Chief Igor Rudan as a Fellow

Established by Royal Charter in 1783 by key proponents of the Scottish Enlightenment, the Royal Society of Edinburgh (RSE) serves as Scottish National Academy that admits Fellows from a wide range of disciplines. The work of the RSE includes awarding research funding, leading on major inquiries, informing public policy and delivering events across Scotland to inspire knowledge and learning. This year, RSE admitted the JoGH’s co–Editor–in–Chief, Prof. Igor Rudan, as a Fellow of the Royal Society of Edinburgh (FRSE).

Prof. Igor Rudan is particularly credited for his contributions to reduction in global child mortality in the 21^st^ century through generating critical evidence that was required for developing successful health policies, and for developing novel methods for prioritizing investments in global health and development that have been widely used by international organizations. In his efforts to reduce global child mortality, he served as a consultant of the World Health Organization, UNICEF, The Bill and Melinda Gates Foundation, The World Bank, Save the Children and others. He also founded the biobank in isolated populations of Croatian islands, which contributed to the discovery of biomedical role for more than 1000 human genes to date. He joined the University of Edinburgh in 2001. He has published 400 research papers and 7 books focused on global maternal and child health and genetic basis of human disease. He has been awarded 20 national and international research awards and professional recognitions.

**Figure:**
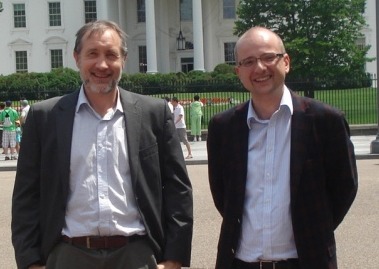
Photo: Prof. Ana Marušić

**Figure:**
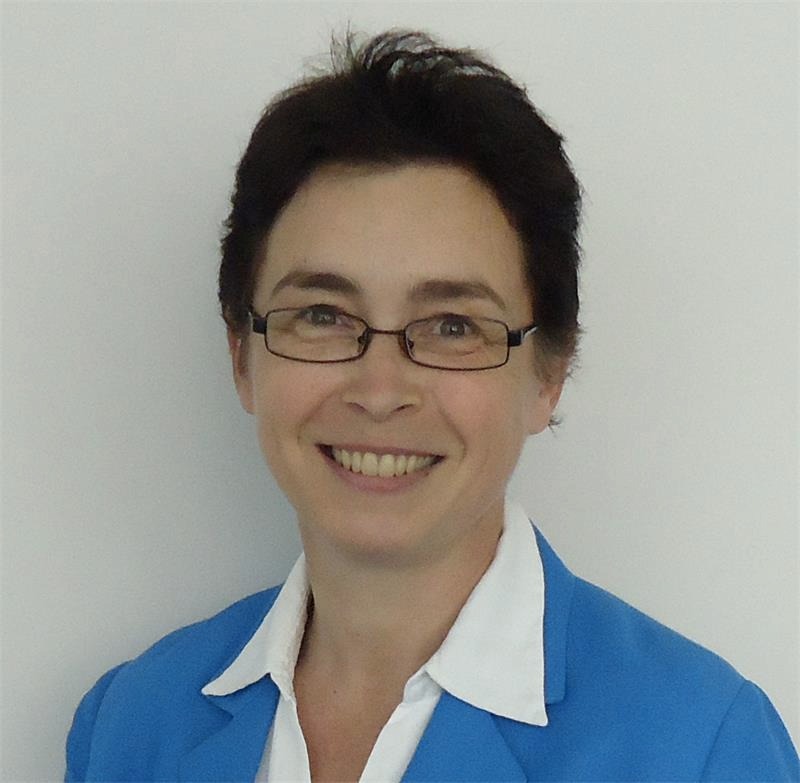
Photo: Prof. Harry Campbell (left) and Prof. Igor Rudan (right)

**Figure:**
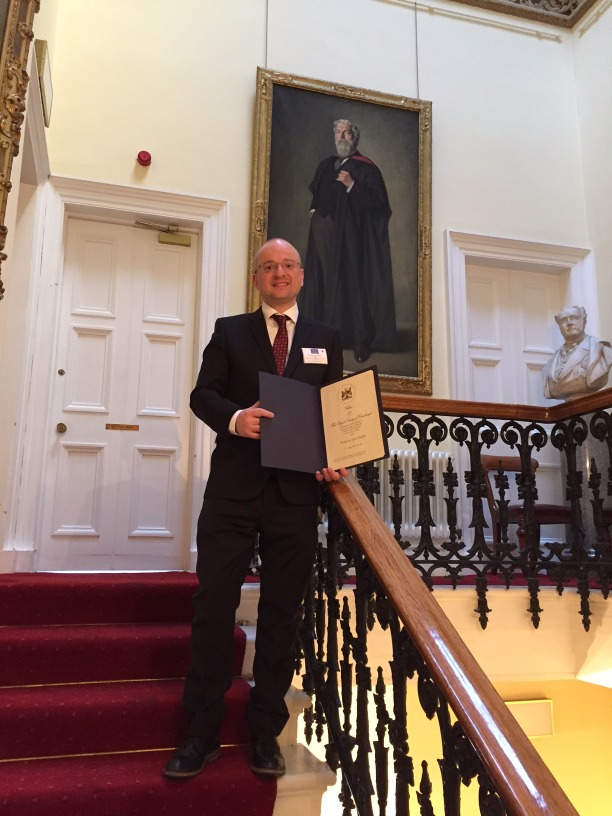
Photo: Prof. Igor Rudan's Fellowship of the Royal Society of Edinburgh

